# P-435. Prevalence and Risk Factors for *Toxoplasma gondii* Seropositivity in a Geographically Defined Cohort of Persons with HIV in Southern Alberta, Canada

**DOI:** 10.1093/ofid/ofae631.635

**Published:** 2025-01-29

**Authors:** Charlotte Rosen, M John Gill, Esther Fujiwara, Hartmut Krentz, Claire Kamaliddin, Hong Yuan Zhou, Jacqueline M McMillan, Brenda Beckthold, Raynell Lang

**Affiliations:** University of Calgary, Calgary, Alberta, Canada; University of Calgary, Calgary, Alberta, Canada; University of Alberta, Edmonton, Alberta, Canada; University of Calgary, Calgary, Alberta, Canada; University of Calgary, Calgary, Alberta, Canada; University of Calgary, Calgary, Alberta, Canada; University of Calgary, Calgary, Alberta, Canada; University of Calgary, Calgary, Alberta, Canada; University of Calgary, Calgary, Alberta, Canada

## Abstract

**Background:**

New evidence suggests *Toxoplasma gondii* infection may have inflammatory and neurocognitive effects. Among people with HIV (PWH) with immunosuppression, *T. gondii* infection can reactivate causing significant morbidity. We aimed to identify the prevalence and risk factors for *T. gondii* infection among a geographically defined cohort of PWH.

**Figure 1:**
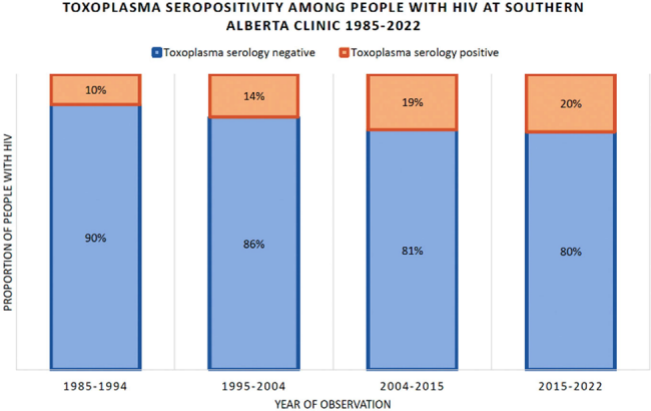
The proportion of Toxoplasma seropositive and seronegative tests among people with HIV at Southern Alberta Clinic

**Methods:**

The Southern Alberta Clinic (SAC) provides centralized HIV care to all PWH in southern Alberta. SAC is linked with a comprehensive database that collects *T. gondii* serology (IgG) on entry into the cohort. Using this database, we evaluated temporal trends and risk factors for *T. gondii* infection between January 1st, 1985 and January 6th, 2022. We used Poisson regression models with robust variance to estimate unadjusted and adjusted prevalence ratios (aPR) with 95% confidence intervals (CI) for factors associated with *T. gondii* seropositivity (vs. seronegativity) among PWH.

**Figure 2:**
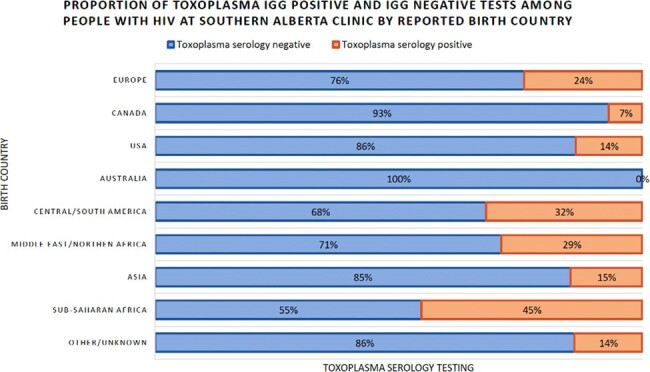
Toxoplasma seropositivity by country of birth among people with HIV at Southern Alberta Clinic from 1985-2022

**Results:**

Among 4,424 PWH receiving care at SAC between 1985-2022, 3,696 (84%) were *T. gondii* IgG negative and 728 (16%) *T. gondii* IgG positive at cohort entry. Over time, the prevalence of *T. gondii* infection has been increasing (Figure 1). In adjusted analyses, *T. gondii* seropositivity was associated with older age >50 years (vs. < 30 years) at serology testing (aPR: 1.7 [95% CI 1.2-2.5]) and birth outside of North America (vs. birth within North America), Central/South America (aPR: 4.5 [95% CI 2.9-6.7]), Sub-Saharan Africa (aPR: 4.3 [95% CI 2.8-6.5]), and Middle East/Northern Africa (aPR: 3.6 [95% CI 2.0-6.4]) (Figure 2).

**Conclusion:**

We found an increasing prevalence of *T. gondii* infection among our population of PWH over time likely associated with a greater proportion of our population being both born outside of North America and age >50 years. Identifying the prevalence and risk factors for *T. gondii* seropositivity is important for monitoring potential negative outcomes associated with *T. gondii* infection and risk stratification among PWH.

**Disclosures:**

**All Authors**: No reported disclosures

